# Genome sequencing and *de novo* assembly of *Photobacterium arenosum* isolate from coastal sediment

**DOI:** 10.1128/mra.01340-24

**Published:** 2025-05-07

**Authors:** Carlos Helbig, Stefan Rasche

**Affiliations:** 1 Department of Plant Biotechnology, Fraunhofer Institute for Molecular Biology and Applied Ecology IME 318347, Aachen, Germany; Montana State University, Bozeman, Montana, USA

**Keywords:** SMRT sequencing, marine microbiology

## Abstract

Genomic analyses have led to the discovery of novel chitinases with emerging applications as environmentally friendly alternatives to synthetic chemical pesticides and for processing renewable resources in various industries. Here, we report the sequencing and assembly of the chitinolytic marine bacterium *Photobacterium arenosum* isolated from coastal sediment from Oostende, Belgium.

## ANNOUNCEMENT

Genomic and transcriptomic analyses have led to the discovery of novel chitinases with unique characteristics, such as different substrate specificities and enzymatic activities brought about by variations in amino acid sequences (1). Chitinases have gained increasing interest for various applications, including the production of chitin-degradation products and as biological control agents for crop protection against pests and fungal phytopathogens (2). Consequently, chitinases have emerged as an environmentally friendly alternative to synthetic chemical pesticides and hold potential for processing renewable resources in various industries (3, 4). *Photobacterium arenosum* is a novel gram-negative, aerobic, chitinolytic marine bacterium (5). *P. arenosum* CAU 1568 was isolated from marine sediment sand sampled at Sido Island in the Republic of Korea (5). In 2022, *P. arenosum* WH24 was isolated from the gill of the oyster *Crassostrea gigas*, caught at the Wilhelmshaven Sea in northern Germany (6). In this study, we report the sequencing and *de novo* assembly of the genome of *P. arenosum* isolated from coastal sediments in Oostende, Belgium.

A seawater sample was transferred to minimal medium with chitin as the sole carbon source. *P. arenosum* was selected as a promising strain with high chitinase activity after three consecutive rounds of enrichment culture (7). Tryptic soy complex medium was inoculated with a *P. arenosum* glycerol stock and cultivated shaking at 28°C for 16 hours. Genomic DNA was isolated from the culture using a NucleoSpin Tissue DNA extraction kit (Macherey-Nagel, Düren, Germany) according to the manufacturer’s instructions. The isolated DNA was purified by AMPure bead cleanup (Beckman Coulter, Brea, USA). Subsequently, a 10 kb PacBio SMRTbell library was prepared with Blue Pippin size selection, following the manufacturer’s instructions. No shearing was performed prior to library preparation. The library was sequenced on a PacBio Sequel SMRT HiFi platform. Data quality check and adapter trimming were performed using SMRT Link v.10.1.0 (PacBio), resulting in 978,480 HiFi reads. The reads were assembled using Flye v.2.9 (8) with the parameters “--pacbio-hifi” and “--genome size 5.5 m”, resulting in six contigs. The contigs were BLASTed on the National Center for Biotechnology Information (NCBI) servers using BLAST v.2.2 (9). The best matches included *P. arenosum* CAU1568 and WH24 and were used for scaffolding using Medusa v.1.6 (10), yielding five scaffolds. The assembly was evaluated using Quast v.5.2.0 (11), polished using BWA-MEM v.0.7.18 (12) and SAMtools v.1.2 (13), for mapping, sorting, and indexing, respectively, and finally corrected by remapping the reads to the assembled genome using Pilon v.1.22 (14). All algorithms were run with default parameters. The draft genome consists of five linear scaffolds with a total size of 4.87 Mbp, mean coverage of 48×, *N*50 of 2,700,155, and guanine-cytosine content of 48.9%. Quality analysis was performed using CheckM v.1.2.3 (15), indicating 87.49% completeness (50th percentile) and 2.09% contamination. The genome was subsequently annotated using the NCBI Prokaryotic Genome Annotation Pipeline v.6.8 (16), predicting 4,462 genes in total (Table 1).

**TABLE 1:** Distribution of CDSs, rRNAs, and tRNAs on scaffolds determined with NCBI Prokaryotic Genome Annotation Pipeline

Scaffold	Protein coding genes	Pseudogene	ncRNA	RNase P RNA	rRNA	tRNA	Other
NZ_JASHHZ010000001.1	2,299	59	1	1	16	71	1
NZ_JASHHZ010000002.1	34	7	0	0	0	0	0
NZ_JASHHZ010000003.1	1,640	34	0	0	0	15	1
NZ_JASHHZ010000004.1	239	11	0	0	7	25	0
NZ_JASHHZ010000005.1	1	0	0	0	0	0	0
Total	4,213	111	1	1	23	111	2

## Data Availability

The assembly results of *Photobacterium arenosum* have been deposited at National Center for Biotechnology Information (NCBI) data sets under accession number GCA_031851305.1. The BioProject accession number is PRJNA973009. The NCBI sequence read archive accession number is SRP561967.
